# Health-Related Quality of Life, Illness Perception, Stigmatization and Optimism Among Hematology Patients: Two Exploratory Path Models

**DOI:** 10.3390/medicina61091704

**Published:** 2025-09-19

**Authors:** Hedvig Kiss, Vanessa Müller, Kristóf Tamás Dani, Bettina Franciska Pikó

**Affiliations:** 1 Department of Behavioral Sciences, Albert Szent-Györgyi Medical School, University of Szeged, 6722 Szeged, Hungary; 2 Department of Hematology, Department of Internal Medicine, Albert Szent-Györgyi Health Center, University of Szeged, 6725 Szeged, Hungary

**Keywords:** health-related quality of life, illness perception, stigmatization, optimism, exploratory path model

## Abstract

*Background and Objectives:* Hematological diseases can cause lasting physical and social impairments. Illness perception and emotional functioning, influenced by stigmatization and optimism, may shape these outcomes, yet their combined effects on health-related quality of life remain underexplored. This study investigates their direct and indirect links with physical and social functioning. *Materials and Methods:* Ninety-six hematology patients completed the EORTC QoL Scale, the Brief Illness Perception Questionnaire, the Stigma Scale for Chronic Illness, and the Revised Life Orientation Test. Analyses were performed using SPSS 25.0 software using descriptive statistics, correlations and path analysis. *Results:* The results indicated that more negative illness perception indirectly affected physical functioning through role (β = 0.38, *p* < 0.01) and cognitive functioning (β = 0.21, *p* < 0.05). Emotional functioning indirectly influenced social functioning via illness perception (β = −0.23, *p* < 0.05) and stigmatization (β = −0.34, *p* < 0.01), moderated by optimism. Additionally, illness perception directly predicted physical functioning (β = −0.21, *p* < 0.05), while emotional functioning directly predicted social functioning (β = −0.37, *p* < 0.01).; *Conclusions:* Illness perception and emotional functioning play a crucial role in shaping physical and social functioning among hematology patients. Optimism moderates these relationships, suggesting that supportive care efforts should target not only improving patients’ perceptions and emotional well-being but also fostering optimism to enhance overall health-related quality of life.

## 1. Introduction

Hematological conditions, ranging from benign disorders like anemia and clotting abnormalities to malignant cancers such as certain types of leukemia, lymphoma, and multiple myeloma, represent a major burden on global health and significantly impact patients’ quality of life [1]. Despite advances in treatment modalities, definitive cures remain elusive for many patients, and long-term disease management is often necessary. Symptoms such as fatigue, nausea, pain, and cognitive impairments are common, leading to significant disruptions in patients’ daily lives, emotional well-being, and social participation. These multifaceted challenges highlight the importance of addressing not only biomedical outcomes but also health-related quality of life (HRQoL) as an essential component of comprehensive patient care. HRQoL in hematology patients is influenced by a complex interplay of physical, psychological, and social factors. Patient-reported outcomes (PROs) have gained increasing attention as vital indicators of treatment effectiveness and patient well-being. Over the past three decades, the European Organisation for Research and Treatment of Cancer (EORTC) Quality of Life Group has developed and validated instruments to systematically assess HRQoL in cancer patients, including those with hematological malignancies [2]. Such tools facilitate a better understanding of how patients experience their disease beyond clinical parameters and support a patient-centered approach to healthcare delivery.

The conceptual framework most often referenced when examining HRQoL is the Wilson and Cleary model [3,4], which links biological and physiological factors to symptoms, functional status, general health perceptions, and overall quality of life. This model has undergone several refinements [5,6], incorporating psychological and social dimensions that align with a biopsychosocial approach to healthcare. Among the five dimensions of HRQoL, physical functioning is the most significantly affected area [7], while social functioning, through several limitations in social life, is the second most affected domain [8]. Although studies indicate improvements in physical and social functions over time, declines were confirmed after chemotherapy [9]. Cognitive functioning such as memory may be less affected in some chronic conditions; however, research notes declines in physical, cognitive, and role functioning in hematology patients [10]. Role functioning is notably affected shortly after treatment but improves significantly in the following years [11]. Within this framework, psychological constructs such as illness perceptions, stigmatization, and optimism have been identified as important contributors to HRQoL outcomes [12,13].

Among these psychological variables, illness perception has emerged as a particularly influential factor [14]. It refers to the cognitive and emotional representations individuals form about their illness, encompassing beliefs about symptoms, causes, consequences, and controllability. Research consistently shows that more adaptive illness perceptions are associated with better HRQoL outcomes [15]. Physical functioning can deteriorate with negative illness perceptions, while perceptions of disease burden can shape preferences regarding social activities resulting in decreased social functioning. Emotional functioning is also closely linked: patients with more adaptive views often manage illness-related emotions more effectively. Role functioning depends partly on beliefs about personal capability, whereas cognitive functioning can suffer if the illness is perceived as an unbeatable threat or source of suffering. Thus, understanding patients’ subjective views of their illness can therefore offer critical insights into tailoring psychosocial interventions to improve HRQoL outcomes.

A special aspect of illness perception is stigmatization, which can particularly worsen HRQoL. While social stigma stems from stigmatizing attitudes towards a person living with illness, self-stigma is the perception of stigmatization within the stigmatized person [16]. Cancer-related stigmatization is an enormous barrier to cancer care, leading to detrimental effects on HRQoL. Previous cancer studies revealed that stigmatization had statistically significant negative associations with all five HRQoL domains [17]. Concerns about stigmatization have also been raised in the field of hematology since some medical interventions and diseases have been associated with higher levels of stigmatization [18,19].

Additionally, optimism, defined as a generalized expectation that good things will happen, has been proposed as a protective psychological factor that can buffer the negative impact of illness-related stressors [20]. Previous research suggests that optimism may not only exert direct effects on well-being but may also moderate the relationship between illness perceptions and HRQoL. For instance, a study by Hurt et al. [21] among patients with Parkinson’s disease found that optimism mitigated the adverse effects of negative illness perceptions on psychological outcomes, highlighting the relevance of optimism as a protective moderating factor. However, relatively little is known about how optimism may moderate the relationships between illness perception, stigmatization, emotional functioning, and HRQoL specifically in hematology patients.

Given these considerations, the present study aimed to investigate the direct and indirect effects of illness perception and emotional functioning on physical and social functioning—two key aspects of HRQoL—in patients with hematological diseases. We further explored the potential moderating role of optimism in these relationships. Two exploratory regression-based path models were developed to examine links between psychological variables and HRQoL dimensions. These two separate models were selected to address the distinct and multifaceted ways that psychological factors impact both physical and social functioning. The first model focused on physical functioning, exploring associations with illness perception, optimism, role, and cognitive functioning. The second model centered on social functioning, incorporating illness perception, optimism, emotional functioning, and stigmatization. This dual approach allows for a nuanced understanding of how different psychological domains affect these two critical aspects of HRQoL, with the goal of informing targeted supportive care interventions.

## 2. Materials and Methods

### 2.1. Study Type

This cross-sectional survey was conducted at a tertiary care health center in southern Hungary from October 2022 to January 2023. Participants were recruited from the hematology department, and eligibility included being 18 years or older, undergoing treatment for a hematological disease, and being physically able to complete the survey. Written informed consent was obtained from all participants.

### 2.2. Sample

As shown in Table 1, a total of 96 adult participants were recruited. Based on previous statistics of the department’s monthly patient turnover and learning from disease statistics, approximately 65% of all patients will be eligible for the aforementioned criteria. Finally, the current sample of 96 patients, representing 94%, that is, a great majority of the target population, admitted to the department during the data collection period, participated in the study. They had a mean age of 56.45 years and 43.8% were female. The ECOG performance status score [22] of the majority of the sample (*n* = 58; 60.4%) was 0, meaning fully active patients who were able to carry on all pre-disease performances without restriction. Diagnoses included leukemia or chronic myeloproliferative disease (54.2%), lymphomas (33.3%), and other rare hematologic diseases (12.5%). Despite the relatively small sample size, the relevance of this study may be supported by another research using a cross-sectional design on 103 non-randomly recruited subjects that tested a path model analysis of depression [23].

### 2.3. Measures

HRQoL was assessed using the validated Hungarian version of the EORTC Quality of Life Questionnaire v3.0. This 30-item questionnaire covers three domains; the present study focused on the functioning domain, including physical, role, cognitive, emotional, and social functioning. Items refer to the past week and are rated on a four-point Likert scale (1–4), with higher scores indicating better functioning. Internal consistency values were acceptable (>0.70) for all subscales except cognitive functioning (0.78 physical, 0.87 role, 0.86 emotional, 0.60 cognitive, 0.80 social functioning), consistent with previous findings [24].

Illness perceptions were measured with the Hungarian version of the Brief Illness Perception Questionnaire [25], comprising eight items. Responses are given on a 0–10 Likert scale, with higher scores indicating more negative illness perceptions (total score range: 0–80). The scale showed a Cronbach’s alpha of 0.64, comparable to earlier studies [26].

Stigmatization was assessed using the Hungarian adaptation of the Stigma Scale for Chronic Illness-8 [27], containing eight items rated from 1 (never) to 4 (always), with total scores ranging from 8 to 40; higher scores reflect greater stigma. Reliability in this study was 0.79.

Optimism was measured with the Hungarian version of the Revised Life Orientation Test [28], which views optimism–pessimism as a bipolar, unidimensional construct, with an equal number of positive and negative items, plus four filler items that are not scored. Responses were provided on a five-point Likert scale (1 = strongly disagree to 5 = strongly agree), with higher scores indicating greater optimism. The total score ranges from 0 to 40, and the reliability coefficient was 0.60, consistent with previous studies [29].

### 2.4. Statistical Analysis

Data were analyzed using SPSS software (Version 25.0). Missing data were excluded using pairwise deletion. Pearson correlation coefficients explored relationships between variables. Multicollinearity was assessed using the Variance Inflation Factor (VIF) with VIF values ≥ 5.0 and tolerance values < 0.2 indicating considerable collinearity. The estimated model for all variables with statistically significant correlations was informed by the theoretical background and bivariate correlation matrix. To ensure the conditions were met for mediation and moderation, the study followed the recommendations of Baron and Kenny [30]. Then, regression-based moderated mediation analyses were conducted using Preacher and Hayes [31] PROCESS macro v3.3 for IBM SPSS (models 7 and 14). In accordance with Hayes’ recommendations [32], bias-corrected 95% confidence intervals were selected. In this case, the indirect effect is significant if the confidence intervals do not include zero. To test significant effects, a bootstrapping method with 5000 bootstrap resamples was used. All variables in the model were standardized to ensure the correct beta weights. Conditional indirect effects were assessed at three distinct levels: one standard deviation above, one standard deviation below the sample mean, and at the sample mean itself. To double-check the convergent validity and enhance the robustness of assessments, CLC Estimator was used, and the congeneric estimation method was applied. Composite reliability (CR) was satisfactory (0.70), and average variance extracted (AVE) values supported convergent validity. A detailed summary of the items alongside their corresponding scale reliability can be found in Table 2.

### 2.5. Ethical Approval

The study was conducted in accordance with the Declaration of Helsinki and approved by the National Scientific and Ethical Committee of the Medical Research Council (ETT-TUKEB, Hungary) (protocol code: #BMEÜ/2299/2022/EKU, issued: 3 October 2022).

## 3. Results

### 3.1. Descriptive Statistics and Bivariate Relationships

Table 3 shows that patients’ illness perception was relatively poor, with no significant stigmatization. Optimism levels were moderate, and all HRQoL subscales averaged around 3.0 (SD < 1.0), indicating a relatively healthy HRQoL. The table also presents alpha reliabilities, means, standard deviations, ranges, minimum and maximum scores, and bivariate correlations for all scales. Pearson correlation revealed statistically significant negative relationships between illness perception and all scales except stigmatization: optimism, physical functioning, role functioning, emotional functioning, cognitive functioning, and social functioning (all *p* < 0.01). Stigmatization was negatively correlated with all function scales: physical (*p* < 0.05), role (*p* < 0.05), emotional (*p* < 0.01), cognitive (*p* < 0.01), and social (*p* < 0.01). Optimism was positively associated with emotional functioning (*p* < 0.05) and illness perception. HRQoL subscales were statistically significantly correlated, especially physical with role functioning and emotional with cognitive functioning (both *p* < 0.01).

### 3.2. Exploratory Path Models

#### 3.2.1. Path Model for Physical Functioning

**Figure 1:**
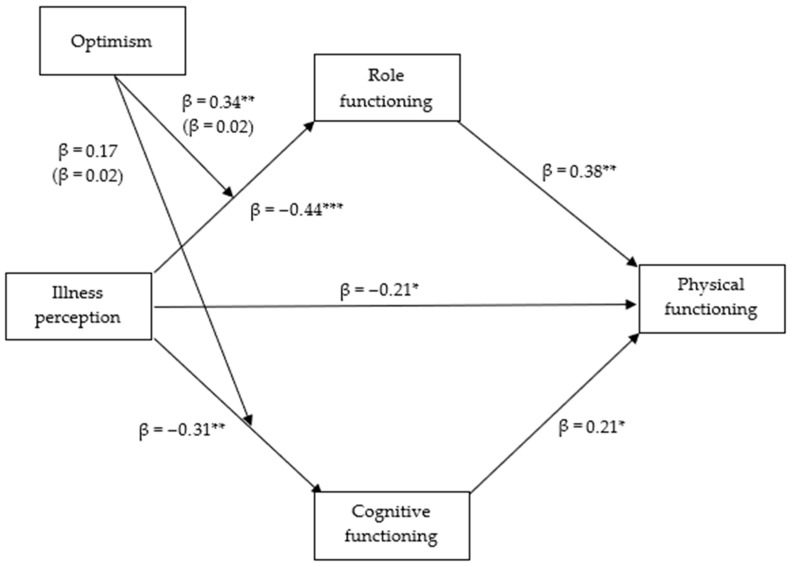
The observed model shows physical functioning as the outcome variable, illness perception as a predictor, cognitive and role functioning as mediators with the moderation of optimism. * *p* < 0.05. ** *p* < 0.01. *** *p* < 0.001.

The first model (Figure 1) showed illness perception negatively impacted role (β = −0.44, *p* < 0.001), physical (β = −0.21, *p* < 0.05), and cognitive functioning (β = −0.31, *p* < 0.01). Conversely, role (β = 0.38, *p* < 0.01) and cognitive functioning (β = 0.21, *p* < 0.05) positively predicted physical functioning. Optimism moderated the effect of illness perception on role functioning (β = 0.34, *p* < 0.01), weakening its negative impact at higher optimism levels. At low (−1 SD) and mean levels of optimism, the conditional effects of illness perception on role functioning are statistically significant, with coefficients of −0.80 (95% CI = −1.12; −0.48) and −0.44 (95% CI = −0.64; −0.24), respectively (both *p* < 0.001). The 95% bias-corrected confidence interval consistently excluded zero; however, this effect diminished (95% CI = −0.39; 0.24) and became statistically non-significant at high levels (+1 SD) (coefficient = −0.08, *p* = 0.63). A moderated mediation effect was found, where the impact of illness perception on physical functioning via role functioning diminished as optimism increased, explaining 43% of the variance in physical functioning (*p* < 0.001). This effect was more pronounced at lower (−1 SD; 95% CI = −0.57; −0.06) and mean levels (95% CI = −0.33; −0.03) of optimism. The indirect effect is illustrated by an index of moderated mediation of 0.13 (CI = 0.02; 0.28). The 95% bias-corrected confidence intervals did not include zero in each case, based on 5000 bootstrap samples. This effect was not statistically significant through the indirect route of cognitive functioning (95% CI = −0.02; 0.11).

#### 3.2.2. Path Model for Social Functioning

**Figure 2:**
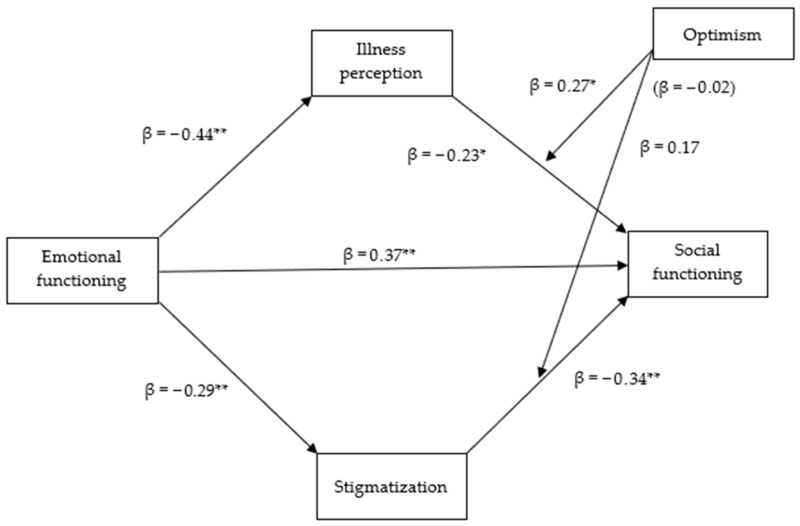
The observed model shows social functioning as the outcome variable, emotional functioning as a predictor, illness perception and stigmatization as mediators with the moderation of optimism. * *p* < 0.05. ** *p* < 0.01.

The second model (Figure 2) revealed positive associations between emotional and social functioning (β = 0.37, *p* < 0.01), and a negative link with illness perception (β = −0.44, *p* < 0.01) and stigmatization (β = −0.29, *p* < 0.01). Stigmatization had a direct negative effect on social functioning (β = −0.34, *p* < 0.01), while illness perception also negatively affected it (β = −0.23, *p* < 0.05). Notably, optimism did not directly predict social functioning but moderated the impact of illness perception. At low (−1 SD; 95% CI = −0.81; −0.17) and mean levels (95% CI = −0.44; −0.15) of optimism, the conditional effects of illness perception on social functioning were statistically significant, with coefficients of −0.51 (*p* < 0.01) and −0.23 (*p* < 0.05), respectively. The percentile bootstrap confidence interval was above zero. However, this was not the case at a high level (+1 SD; 95% CI = −0.27; 0.34), suggesting that among highly optimistic individuals, illness perception does not statistically significantly affect social functioning. The model showed a moderated mediation effect, with the impact of emotional functioning on social functioning through illness perception, with an index of moderated mediation of −0.12 (95% CI = −0.26; −0.03). This effect was statistically significant at low levels (−1 SD) of optimism (B = −0.022, 95% CI = 0.08; 0.42) and to a lesser extent at the mean level (B = −0.10, 95% CI = 0.02; 0.19). The direct and indirect paths were statistically significant, as the 95% bias-corrected confidence intervals did not include zero in each case. Nonetheless, this indirect effect was not statistically significant at high levels (+1 SD; 95% CI = −0.18; 0.08) of optimism, indicating that the negative impact of illness perception on emotional and social functioning was stronger at lower levels of optimism. The overall path model explained 49% of the variance in social functioning scores (*p* < 0.001).

## 4. Discussion

This study aimed to explore the interconnectedness of various aspects of HRQoL among Hungarian hematology patients and their association with psychological factors, using exploratory path models.

Participants reported relatively poor illness perception, a finding consistent with existing literature [33]. Lekarakou, Koulierakis and Pontikoglou [34] highlighted the importance of improving disease management and appropriate interventions, as patients’ dramatic narratives, feelings of distress, anxiety and depression levels, fatigue, and psychological well-being are key components of their subjective illness perceptions. While stigmatization levels were generally low, felt stigma was found to have a more disruptive impact than enacted stigma [35]. Moderate levels of optimism were observed in the sample, and HRQoL subscales suggested generally good QoL. However, hematological diseases often negatively affect HRQoL, particularly in physical and emotional dimensions [36].

Illness perception had statistically significant negative correlations with optimism and all HRQoL subscales, except stigmatization, which negatively impacted all HRQoL function scales, consistent with prior research [37]. Optimism was positively associated with emotional functioning and negatively with illness perception, aligning with previous findings [38]. All HRQoL subscales were positively correlated, confirming their interconnectedness. Thus, poorer illness perception and higher levels of stigmatization were linked to reduced HRQoL, while optimism acted as a protective factor.

The first model highlighted the direct impact of illness perception on physical functioning, with cognitive and role functioning as mediators, and optimism as a moderator. Subjectively poor illness perception, coupled with less adaptive coping strategies and declined functional abilities across roles, has been previously shown to impair HRQoL [39]. Prior studies suggest that negative illness perceptions—such as greater concern over disease severity and perceiving it as debilitating—lead to diminished physical functioning [33]. Optimism mitigated the negative effects of poor illness perceptions on physical functioning [14]. Illness perception also had a statistically significant negative relationship with role and cognitive functioning, supporting the idea that poor illness perception can impair these areas [40]. Westbrook et al.’s [41] data further support this, suggesting that the emotional and cognitive representation of illness shapes beliefs about the ‘health threat’ (e.g., consequences or controllability), affecting both psychological and physical outcomes. The feeling of having a limiting health condition can interfere with cognitive functions like memory, exaggerating the severity or frequency of symptoms. Role and cognitive functioning positively predicted physical functioning, indicating that better cognitive capabilities and social engagement reduce physical limitations. This suggests that role and cognitive functioning may enhance physical functioning, despite negative illness perceptions.

In contrast to previous studies [13], optimism emerged as a crucial moderator rather than having a direct connection with HRQoL. One study [42] examining the mediation and moderation of optimism found it to be a mediator in the pain–QoL relationship; while another [43] depicted optimism as a moderator in the relationship between social support and emotional well-being. In our study, optimism moderated particularly the link between illness perception and role functioning. The moderated mediation effect revealed that optimism reduced the impact of illness perception on role functioning, a key predictor of physical functioning. This finding underscores the pivotal role of optimism in recognizing one’s active role in therapy and aligns with the positive psychology model. Our data suggest that the effect of illness perception on role functioning is most pronounced in less optimistic patients, highlighting that poorer illness perception acts as a barrier to appropriate physical functioning through inadequate role functioning.

The second model revealed a direct link between emotional and social functioning, consistent with existing literature [9]. Hematological diseases can lead to emotional challenges that impact social engagement [44]. Emotional functioning showed a strong negative association with illness perception and stigmatization, influencing social interactions. Consistent with prior findings [17], a statistically significant negative relationship was found between illness perception, stigmatization, and social functioning. Illness perception and stigmatization acted as mediators, with optimism moderating their impact on social functioning, particularly among less optimistic patients. A recent study [45] has also described the mediating role of illness perception, indicating that social constraints affect HRQoL through illness perceptions, fear of relapse, and the combined mediating effect of both. In our study, optimism weakened the impact of illness perception on social functioning, especially in patients with lower optimism levels. Emotional functioning naturally suffers upon diagnosis of a serious illness, potentially leading to distress or anger. These emotional responses can influence illness perception and social interactions. The moderation effect of optimism was more pronounced in those with lower optimism, indicating that patients who are emotionally less stable might perceive their illness more negatively, impacting their social functioning.

This study contributes to the literature by focusing on Hungarian hematology patients and examining HRQoL in a dimensional way. A novel aspect is the focus on two dimensions—physical and social functioning—which are among the most important in the literature, emphasizing their interrelationships. The findings highlight the importance of optimism in managing illness and its impact on daily life. Clinical implications include the need for tailored interventions targeting both physical and emotional aspects of illness, and enhanced communication training for healthcare professionals to better support patients’ emotional well-being. Healthcare providers should be educated about HRQoL and its dimensions to better understand which aspects matter most to each patient, allowing for personalized care. Developing practical courses on holistic patient care could help healthcare professionals improve HRQoL by making them more aware of the importance and interconnections between HRQoL dimensions. In the context of the patient population, offering educational materials that focus on managing emotional issues related to disease, such as stigmatization, could be crucial.

The study has limitations. Its cross-sectional design limits causal inferences, and the small sample size restricts generalizability. Data were collected from patients with sufficient physical and cognitive abilities, and the study relied on self-reported data. In addition, the relationships between the examined variables can be more complex depending on patients’ illness characteristics. Another limitation is that information on marital status was not collected, although it may be an important factor influencing optimism and overall HRQoL. Furthermore, due to the limited sample size, we were not able to stratify patients by disease type or age group, although different diseases and age groups may be associated with distinct illness perceptions. Longitudinal studies with larger, more diverse populations could provide a more comprehensive view of the relationships between these variables and enhance generalizability. Combining quantitative and qualitative methods could offer deeper insights, and further research into factors such as social support could help clarify the role of psychosocial variables in HRQoL. Finally, some scales need further validation due to relatively low reliability values, ensuring robustness and accuracy of the measures used in this research. Nevertheless, as the first study examining HRQoL in hematology patients in Hungary, these findings highlight the significant associations between illness perception and its correlates.

## 5. Conclusions

In conclusion, the study achieved its aim of exploring the psychological determinants of health-related quality of life in detail in patients with hematological diseases. Illness perception and emotional functioning were identified as key determinants of HRQoL, while optimism appeared to moderate these relationships. By developing two separate path models, we demonstrated that psychological variables influence physical and social functioning in distinct ways. Applying this dual approach represents a novelty of this paper, as it enables the identification of nuanced differences in the path structure of various dimensions of HRQoL. The current sample adds further novelty, given that only a few studies have included patients with hematological diseases while examining different dimensions of HRQoL. Furthermore, the inclusion of both favorable (e.g., optimism) and unfavorable (e.g., stigmatization) psychological variables also contributes to the originality of our study.

These results highlight the importance of integrating psychosocial support into routine hematology care. Targeted interventions aimed at improving emotional well-being and addressing maladaptive illness beliefs may enhance patients’ physical, emotional, and social functioning, ultimately contributing to better overall quality of life. Multidisciplinary supportive care strategies that promote optimism and adaptive coping could therefore play a critical role in comprehensive cancer care.

## Figures and Tables

**Table 1:** Participants’ demographic and clinical characteristics (*n* = 96).

Variable		*n* (%)	Mean (SD) Median/Range Min–Max
Biological sex			
	Female	54 (56.3)	
	Male	42 (43.8)	
Age (years)			56.45 (15.55) 62/62 21–83
	18–49	29 (30.2)	
	50–64	30 (31.3)	
	65–	37 (38.5)	
Educational status			
	Primary education	35 (36.5)	
	Secondary education	36 (37.5)	
	Tertiary education or higher	25 (26.0)	
Permanent residence (number of residents)			
	Village or smaller (0–5000)	26 (27.1)	
	Small town (5–20,000)	23 (24.1)	
	Medium town (20–100,000)	25 (26.1)	
	Large city (>100,000)	22 (22.9)	
Socioeconomic status			
	Lower and lower middle class	32 (33.3)	
	Middle class	55 (57.3)	
	Upper middle and upper class	9 (9.4)	
Type of disease			
	Leukemias and chronic myeloproliferative disease	52 (54.2)	
	Lymphomas	32 (33.3)	
	Multiple myeloma	9 (9.4)	
	Other non-malignant diseases (ET, PNH, TTP)	3 (3.1)	
Health condition (ECOG status 0–5)			0.48 (0.69) 0–2
	0—fully active, able to carry on all pre-disease performance without restriction	58 (60.4)	
	1—Restricted in physically strenuous activity but ambulatory and able to carry out work of a light or sedentary nature, e.g., light housework, office work	30 (31.3)	
	2—Ambulatory and capable of all selfcare but unable to carry out any work activities; up and about more than 50% of waking hours	8 (8.3)	

Note. Definitions of abbreviations: ET = essential thrombocytosis; TTP = thrombotic microangiopathy; PNH = paroxysmal nocturnal hemoglobinuria.

**Table 2:** Items and reliability of latent variables (congeneric estimation).

Construct and Item Description	Loading	α	CR	AVE
Illness perception	–	0.64	0.91	0.60
IP1	0.78			
IP2	0.94			
IP3	0.80			
IP4	0.73			
IP5	0.59			
IP6	0.81			
IP7	0.56			
IP8	0.86			
Stigmatization	–	0.75	0.89	0.54
STIG1	0.87			
STIG2	0.99			
STIG3	0.82			
STIG4	0.62			
STIG5	0.89			
STIG6	0.40			
STIG7	0.59			
STIG8	0.48			
Optimism	–	0.72	0.90	0.51
OPT1	0.65			
OPT2	0.74			
OPT3	0.69			
OPT4	0.62			
OPT5	0.58			
OPT6	0.88			
OPT7	0.78			
OPT8	0.69			
OPT9	0.82			
OPT10	0.58			
Physical functioning	–	0.78	0.91	0.69
PH1	0.75			
PH2	0.83			
PH3	0.92			
PH4	0.61			
PH5	0.98			
Role functioning	–	0.87	0.74	0.59
R1	0.99			
R2	0.89			
Emotional functioning	–	0.85	0.74	0.58
EF1	0.76			
EF2	0.78			
EF3	0.82			
EF4	0.68			
Cognitive functioning	–	0.61	0.79	0.65
CF1	0.98			
CF2	0.60			
Social functioning	–	0.79	0.73	0.59
SF1	0.88			
SF1	0.99			

Note. Definitions of abbreviations: CR = composite reliability; AVE = average variance extracted; IP = illness perception; STIG = stigmatization; OPT = optimism; PH = physical functioning; R = role functioning; EF = emotional functioning; CF = cognitive functioning; SF = social functioning.

**Table 3:** Correlation matrix of the relationship between the study variables.

Variable	Mean (SD)	Range (Min–Max)	1.	2.	3.	4.	5.	6.	7.	8.
1. Illness perception	40.52 (11.86)	57 (10–67)	(0.64)							
2. Stigmatization	10.87 (3.49)	21 (8–29)	0.19	(0.79)						
3. Optimism	37.05 (4.75)	23 (26–49)	−0.29 **	−0.05	(0.60)					
4. Physical functioning	3.38 (0.602)	3 (1–4)	−0.46 **	−0.26 *	0.23 *	(0.78)				
5. Role functioning	3.20 (0.98)	3 (1–4)	−0.42 **	−0.23 *	0.08	0.63 **	(0.87)			
6. Emotional functioning	3.13 (0.69)	3 (1–4)	−0.48 **	−0.32 **	0.33 *	0.40 **	0.44 **	(0.86)		
7. Cognitive functioning	3.54 (0.66)	3 (1–4)	−0.29 **	−0.49 **	0.10	0.44 **	0.45 **	0.62 **	(0.60)	
8. Social functioning	3.13 (0.95)	3 (1–4)	−0.41 **	−0.46 **	0.10	0.34 **	0.51 **	0.58 **	0.46 **	(0.80)

Note. Alpha values on the diagonal and correlation coefficients above the diagonal. Probability notes for *p* values: * *p* < 0.05. ** *p* < 0.01

## Data Availability

The datasets generated and analyzed during the current study have not been deposited into a publicly available repository. Data will be made available on request.
